# No legal personhood for AI

**DOI:** 10.1016/j.patter.2023.100861

**Published:** 2023-11-10

**Authors:** Brandeis Marshall

**Affiliations:** 1 DataedX Group, Smyrna, GA, USA

## Abstract

As AI technologies grow to encompass more human-like generative capabilities, discussions have begun regarding how and when AIs may merit moral consideration or even civil rights. Brandeis Marshall argues that these discussions are premature and that we should focus first on building a social framework for AI use that protects the civil rights of all humans impacted by AI.

## Main text

Generative AI has accelerated the discussion around whether or not AI does, could, or should have civil rights. As scientists, we are asked to weigh in on such discussions without adequate grounding of the legal implications of our responses. We adopt a mindset that since we are trained in data, algorithms, and AI concepts we are equipped to discuss the intersection of data and AI applications with law and society. This mindset is crafted by tech culture’s “move fast and break things” philosophy that champions speedy calculated risk and tech-styled evolution, where nearly everything is a derivation of tech. “Move fast and break things” has become so entrenched in how we design, implement, and maintain digital systems. Moving fast and breaking things is normalized and accepted behavior when in practice, very few situations in society are conducted at this speed or scale. AI, in recent years, has been elevated toward the top of the social hierarchy. It has therefore become the prime next candidate to impose the “move fast and break things” philosophy by pushing for AI to be afforded some form of legal person rights. But this 30-years-in-the-making global conditioning that tech is by default trustworthy and superior has been more publicly and regularly challenged with increasing pressure for the tech industry to address existing algorithmic-based harms. And the calls for legal personhood for AI must be met with an equal dose of scrutiny.

Civil rights for AI require an understanding first if AI, generative or otherwise, should be designated a legal person. A legal person, as described by US law, is a quantifying designation that refers to a human being or non-human entity that is treated as a person with limiting applications. Legal personhood variants have already been granted to nature, animals, and corporations. Some call for an extension of similar legally binding rights to be granted to AI systems, tools, and platforms.^1^ Others, like me, warn against such allowances for AI. This commentary has a clear stance—as prominently stated with its title. But whether you agree with that stance or not, it’s hopeful that instead of pondering potential AI rights legislation, this’ll spark deeper and robust actions on how we focus on civil rights for humans impacted by AI.

### AI is more pattern recognition than logic and reasoning

AI is commonly described as a computer program or series of computer programs that enable the mimicking of human behavior. An important but frequently overlooked condition is that AI is a docile yet mutable software that is built and adapted by people. AI feeds on data, i.e., content, which in turn fuels the algorithms and statistical models driving automation practices. We need to better unpack and explain the seemingly magical blackbox of AI.

First, we should better understand data, i.e., content. Data and content are interchangeable. Data is the term most scientists and STEM professionals tend to use while content is most frequently used by non-STEM professionals. Data include text, images, audio, video, and other forms of data inputs (e.g., taste, sight, hearing, smell, and touch). Digitizing all data types has proven very difficult, although capturing sound has seen the most consistent advancements. The amount of data, in all its different forms, has varying degrees of accuracy. There’s content based in fact (what we want), misleading content (misinformation), and malicious content (disinformation). Distinguishing among fact, misinformation, and disinformation is yet another hurdle that people have yet to overcome, so AI stalls by halluncinating as a result (see scholarly AI hallucination research^2^^,^^3^^,^^4^).

Second, we should better embrace the reality of what AI is. AI, at its foundational level, is a software system. It is trying to decipher the multiple forms of data using algorithms and statistical models in order to predict outputs consistently. The need for more data helps AI fine-tune algorithmic and statistical model performance until perceived fact-based data have replaced any misleading and malicious data or human behavior doesn’t align with an algorithm’s mathematical equation. We’ve already witnessed AI fabricating content to harmful effect such as a legal brief having fake citations^5^ or a professor being wrongly accused of sexual assault.^6^

And third, we should better accept how AI operates. People have shaped AI to adhere to a single objective, which is to identify and follow the pattern it has provided as input and produce an output. In accomplishing this goal, AI’s limitations are ever more brazen. The inputs and outputs of AI aren’t guaranteed to be accurate. AI is allowed to be mutable in order to make false content. But on the other hand, AI remains docile as people control when AI routines are started and terminated. Most strikingly, AI has yet to provide evidence that it has a moral compass. AI lacks contextual awareness, conflict resolution, and critical thinking. This is evidenced by the AI-generated legal brief instance, mentioned above, that didn’t have the contextual awareness to indicate a lack of legal precedent to finish the legal brief. Rather, this conflict was ignored, not resolved. And the critical thinking needed to suggest alternative legal arguments wasn’t explored. Deductive and inductive reasoning, making inferences, and accurately vetting valid and invalid arguments aren’t part of AI’s construction. To be deemed a person, in the eyes of the law, presupposes that there’s an independent set of personal interests, ability to exercise its own agency, and accountability for their conduct. AI has none of these characteristics because these aspects can’t be effectively digitized.

### Legal personhood assumes personhood, particularly of human beings, is a monolith; It is not

AI as a rights-bearing entity has skipped over a myriad of social, cultural, economic, political, and legal disparities incurred by actual human beings. We should be contextualizing race, gender, class, disability, or other -isms with respect to both our physical and digital spaces. Not everyone in the United States and globally has fully executed civil rights. Inequality and inequity are baked into many governmental systems. For instance, the 1787 Three-Fifths Compromise was political and economic oppression for enslaved Africans wrapped in political and economic power acquisition for white slaveowners. Political oppression was partly abated for Black people in 1870 when the 15th Amendment ratified that Black men were granted the right to vote even though Black people were only recognized as US citizens as part of the 14th Amendment in 1868. White women were granted the right to vote through the 19th Amendment in 1920 while Black women were disenfranchised from voting with a prerequisite literacy test and other discriminatory practices until the 1965 Voting Rights Act. It is important to note that financial reparations for the enslavement of Black people in the United States has yet to become an act or amendment. The State of California, however, has pushed state legislation forward in recent years.

The civil rights’ progress of the 1800s and 1900s are being eroded in the 2000s. In 2022 and 2023, reproductive agency, affirmative action considerations in higher education admissions, and personhood protections were stuck down by the US Supreme Court for birthing people and women and people from historically excluded groups (race, ethnicity, LGBTQ+ identities, etc.). It is clear that being a human being doesn’t guarantee legal personhood protections in all areas of civil rights for some groups. For birthing people, you have been granted the civil right to vote, but you aren’t able to receive the civil right to certain medical care. But if you’re a person not from a historically excluded group or not a birthing person, then your civil rights and personhood remain unaffected and protected. US legal personhood status and realization of civil rights, especially of human beings, has proven to not be a monolith. Our historical recap of the inequitable execution of civil rights reinforces the forced social structure that is compounded by intertwined political, economic, and legal factors.

The same law can have disparate impacts on different groups of people. For example, in the case of child adoption, same-sex couples may face restrictions based on marital status and the US state law prohibitions for LGBTQ+ people while heterosexual couples don’t face the same obstacles. The foundation of imbalance in US law sits at the crux of multiple tensions for historically excluded groups. Prioritizing the most vulnerable populations by law helps to mitigate these tensions and harms while spotlighting unearned privileges. The skewed scales of legal personhood for all human beings need to be remedied first since, as history has shown us, technological innovations mirror our physical society. If we don’t recognize and fulfill the promise of comprehensive civil rights for all human beings, we are on track to further replicate those disparities in the law. We the people aren’t a monolith and nor should we be treated as such. We also shouldn’t enable any lines of code to limit us to operate as a monolith.

The recommendations involve providing remedies that rectify the disparities with respect to full human civil rights under the law and instituting an AI responsibility framework that keeps humans in the loop. Executing the full civil rights of all human beings provides more equitable conditions for a blueprint for AI personhood in the law to exist. This blueprint for AI personhood could then better contextualize the racial, gendered, political, economic, and cultural disparate impacts. We’d be in a more informed position to assess whether pursuing AI personhood rights legislation makes sense.

Since we haven’t figured out how to fully execute human civil rights, it seems premature to have AI personhood conversations. AI, if it were to have legal personhood status and be granted a form of civil rights, would need to address the established social structure along with the political, economic, and legal consequences. Those who operate AI have ignored confronting this structure and its ramifications. Here are three open challenges: (1) AI’s impact to systems, tools, platforms, and institutions has yet to be assessed. (2) The full scale of baked-in AI biases and inequitable power dynamics has yet to be acknowledged, accepted, or atoned. (3) AI has yet to be regulated in order to provide agile guardrails and compliance protocols. An AI responsibility framework, discussed below, can help us identify and assess the degree to which people and lines of code can close gaps, mitigate biases, and combat disparate harms posed by AI systems, tools, and platforms.

### Establishing effective data/AI transparency, accountability, and governance organization first *would* help in protecting people in an AI-filled society

AI, first, should have an established social structure. There should be more formalized methods for protective approaches to prioritizing legal personhood of human beings and preventative strategies related to political-, economic-, and legal-induced harms. Protective approaches could operationalize more transparency of AI use. The AI Dependency Spectrum^7^ is a framework that delineates the amount of AI used to produce the output: AI-generated classification (>90% AI dependent), AI-assisted classification (50%–90% AI dependent), AI-enhanced classification (10%–49% AI dependent), and AI-lite classification (<10% AI dependent). Also, there should be an establishment of a human-created, certified content stamp for tasks that fall in the AI-lite category. The vetting of outcomes wouldn’t be the sole individual responsibility of the recipient. It would primarily be placed on the AI user.

Preventative strategies to combat a myriad of political, economic, and social harms could be executed more by the law. We would need to expand the implementation of punitive consequences for human harmful behavior of AI such as penalties, sanctions, algorithmic destruction,^8^ and human-driven oversight/compliance processes. If the AI industry wants to treat AI as human, then the industry should be subject to penalties experienced by human beings who violate US laws. I explored this approach in *Data Conscience*^9^ by recommending the

[creation of] a legal framework for tech probationary jail, short-term banning, and long-term tech incarceration for algorithms, processes, systems, and tools. Certain technologies need to be sidelined until their impact on society and its people can be determined. Place certain algorithms, processes, and tools in a virtual timeout where their functionalities are disabled and inoperable. This conduct is applied on social platforms when accounts/profiles are marked as incendiary. The same rules need to apply to tech products.

This would disincentivize heavy AI dependency use, e.g., AI-generated and AI-assisted, while encouraging equitable practices and human-driven decision-making competencies.^10^

For AI to achieve this established social structure by having effective data/AI transparency, accountability, and governance, it should legally take at least as long as Black women’s uninhibited right to vote in the United States (346 years = 1965 − 1619).
